# Environmental prediction, risk assessment and extreme events: adaptation strategies for the developing world

**DOI:** 10.1098/rsta.2011.0160

**Published:** 2011-12-13

**Authors:** Peter J. Webster, Jun Jian

**Affiliations:** 1 School of Earth and Atmospheric Sciences, Georgia Institute of Technology, Atlanta, GA, USA; 2 Navigation College, Dalian Maritime University, Dalian 116026, People's Republic of China

**Keywords:** uncertainty, climate, adaption

## Abstract

The uncertainty associated with predicting extreme weather events has serious implications for the developing world, owing to the greater societal vulnerability to such events. Continual exposure to unanticipated extreme events is a contributing factor for the descent into perpetual and structural rural poverty. We provide two examples of how probabilistic environmental prediction of extreme weather events can support dynamic adaptation. In the current climate era, we describe how short-term flood forecasts have been developed and implemented in Bangladesh. Forecasts of impending floods with horizons of 10 days are used to change agricultural practices and planning, store food and household items and evacuate those in peril. For the first time in Bangladesh, floods were anticipated in 2007 and 2008, with broad actions taking place in advance of the floods, grossing agricultural and household savings measured in units of annual income. We argue that probabilistic environmental forecasts disseminated to an informed user community can reduce poverty caused by exposure to unanticipated extreme events. Second, it is also realized that not all decisions in the future can be made at the village level and that grand plans for water resource management require extensive planning and funding. Based on imperfect models and scenarios of economic and population growth, we further suggest that flood frequency and intensity will increase in the Ganges, Brahmaputra and Yangtze catchments as greenhouse-gas concentrations increase. However, irrespective of the climate-change scenario chosen, the availability of fresh water in the latter half of the twenty-first century seems to be dominated by population increases that far outweigh climate-change effects. Paradoxically, fresh water availability may become more critical if there is no climate change.

## 1. Introduction

One of the great challenges of weather and climate science is estimating the probability of the occurrence, severity and duration of an extreme event, as well as its severity and duration, and when and where the event will take place. Tropical cyclones, prolonged droughts and flooding are extreme environmental events that can invoke severe economic cost, societal disruption, death and destruction. Advanced warning of an extreme event allows for preparation, possible evacuation and the marshalling of emergency systems and personnel to help in the mitigation of its effects.

This paper addresses the application of ensemble weather and climate model simulations to aid the less-developed world to adapt to extreme events and climate change. In the more-developed world, solid infrastructure and economic robustness allows for the impact of extreme events to be absorbed by the larger national community through hedging, insurance and/or government support. In the United States, for example, regions impacted by floods and hurricanes may be declared as ‘federal disaster regions’, allowing communities to receive rapid fiscal and personnel aid from government and non-government organizations. Cuaresma *et al.* [1] even argue that a natural disaster in a developed country may provide ‘*constructive destruction*’ where destroyed infrastructure is replaced by more modern artefacts. However, the developing world has generally far fewer options [2].

**Figure 1.**
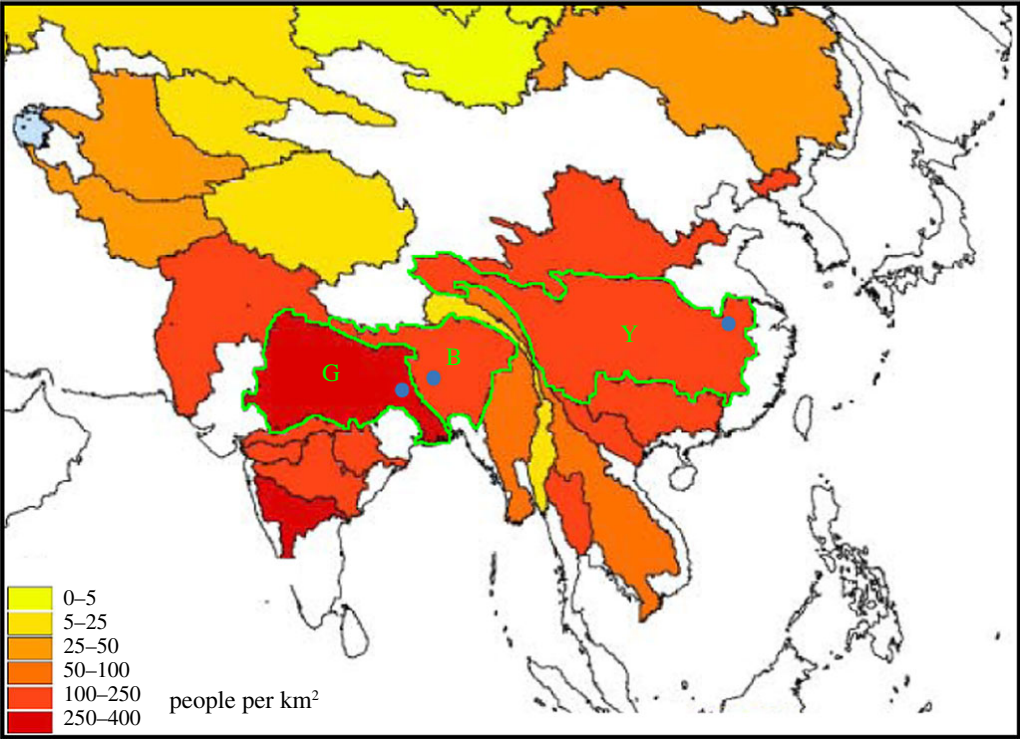
The Himalayas and the Tibetan Plateau are the sources for a complex of major rivers associated with thriving agricultural societies, colour coded by population density. The Ganges (G), the Brahmaputra (B) and the Yangtze (Y) are outlined. These deltas are currently the home to 14% of the human population on the planet. Three river staging stations, the Hardinge Bridge on the Ganges, Bahadurabad on the Brahmaputra and Datong on the Yangtze are shown as blue dots.

Developing countries in South and East Asia (figure 1) are especially vulnerable to extreme weather and climate events, particularly from floods and tropical cyclones. However, these nations often lack the fiscal resilience and the infrastructure to minimize the impacts of extreme events or to be able to recover from them quickly. Devastating floods or a land-falling tropical cyclone may lead to nation-wide fiscal problems and perhaps accelerants of political unrest [3,4]. At the community or village level, the impact is even more severe, forcing many into transient poverty when income is temporally less than expenses [5]. Exposure to multiple extreme events can transform temporary poverty into structural and endemic poverty and possibly perpetual intergenerational poverty [5].

Environmental catastrophes produce great and sudden harm and occur sufficiently irregularly to make preparations difficult. Catastrophes are different from static and perpetual natural hazards such as the arsenic contamination of artesian water in Bangladesh and in other Asian countries [6,7]. For these hazards, relatively inexpensive technological solutions are available for groundwater contamination through the distribution of effective filters (e.g. [8]), requiring merely the will, training and finances. Engineering solutions to counter flood and tropical cyclone surges, on the other hand, are expensive and often even beyond the resources of developed countries. However, if the catastrophic event can be forecast then, mitigatory actions can be undertaken to reduce its impact. The longer the lead time of a warning of an impending event and the greater the specificity of the forecasts, the greater the chances are of protecting lives, property and resources.

Consider the short-term impacts of extreme events in Bangladesh, located at the apex of the Bay of Bengal on the delta of the Ganges and the Brahmaputra rivers (figure 1). Bangladesh had a population of about 160 million in 2009 within an area the size of England [9]. Although great strides have been made in reducing birth rate, it still stands at 2.5 per cent per year, providing a population doubling time of about 30–40 years. The location of Bangladesh makes it especially susceptible to tropical cyclones and flooding. During the summer monsoon period (June–September), slow-rise floods inundate large parts of India and Bangladesh, affecting over 40 million people each year. In India, an average of 60×10^3^ km^2^ of land (approximately equivalent to the size of Texas, Ireland or Norway) is flooded annually [10,11], with an additional 20×10^3^ km^2^ in Bangladesh. In each country, the consequences of flooding are devastating and endlessly impoverishing. Each year before planting, farmers borrow against potential income to be earned at the end of a successful season. These loans are used for future agricultural inputs (seed, fertilizer, pesticides, equipment, stock). The loss of a crop or stock animals during a flood period typically puts a farmer in debt for many years, by which time the cycle of flooding is repeated, condemning the next generation to lives of poverty in repaying debt. Floods are not restricted to the summer. During spring, short-lived deluges associated with pre-monsoon thunderstorms and mesoscale convective events create flash floods that destroy large areas of winter rice [12].

In 1987, 1988 and 1998, extensive flooding occurred throughout Bangladesh when both the Ganges and the Brahmaputra crested simultaneously well above their flood levels. It is estimated that in 1988 and 1989, 3000 people lost their lives, homes of millions were destroyed and over 200 000 cattle drowned. In 1998, over two-thirds of the country was submerged for 3 months, and an estimated 1000 people drowned, with millions left homeless [13,14]. In 2004, 2007 and 2008, shorter term flooding lasting 10 days or so occurred along the Brahmaputra. Although not as devastating as the earlier prolonged floods, these widespread inundations impacted millions of people. Societal vulnerability had increased during the previous decade owing to the rapidly growing population (e.g. [15]) that has forced many people to farm the fertile chars (river islands) prone to chronic flooding, and even disappearance, during a flood.

The location of Bangladesh at the head of the Bay of Bengal also makes it especially susceptible to land-falling tropical cyclones during the boreal spring and autumn. Most loss of life and damage is associated with wind-driven storm surges that often inundate areas of tens of kilometres of the flat delta. In fact, the Bay of Bengal is home to 7 of the 10 most deadly tropical cyclones in recorded history.

This paper provides two examples from the South Asia region whereby anticipation of extreme events through probabilistic weather and climate forecasts could enable a user group to minimize their adverse impacts. Two different time scales are considered: the time scale of days to weeks, with regard to a specific impending extreme event; and the time scale of decades, with regard to the changing statistics of extreme events that may attend climate change. The first task addresses what can be done with present environmental weather and climate models to allow adaptation and minimization of loss. We use, as an example, the immediate problem of providing forecasts of river delta flooding in river deltas on time scales that allow agricultural adaptation, minimization of property loss and evacuation. We also discuss the manner in which these forecasts can be communicated directly to those that will benefit most from advanced warning. We provide examples in §3 of the economic benefit of accurate and timely short-term (1–10 days) forecasts at the community level. In a region anticipating long-term climate change and a possibility of increased frequency of extreme events, long-term planning is required, which may require the building of dams, dykes and river diversions. With a background of rapidly growing population, there is the important issue of ensuring food security, fresh water availability and energy production. An example is provided using climate models in a range of economic and demographic scenarios of the twenty-first century to assess risk of future floods and fresh water availability in South and East Asia.

## 2. Determining the risk of an extreme event

In order to adapt to the impacts of an extreme weather event through the adoption of some strategy (e.g. choosing a drought-resistant crop, plant or harvest early or later) or reduce its impacts (e.g. change water resource management strategies), it is necessary to determine the likelihood of an extreme event occurring in a particular location and time. This can be accomplished from historical data, experience and intuition, and quantitative probabilistic weather and climate forecasts.

Managing weather risk may be thought of as a game of roulette [16,17] in which an event is forecast by the ball falling on either a red or black slot. With no information, there is an equal likelihood of the ball falling into a red or black slot (ignoring the double zero slot). If it is known that slow-rise floods only occur in a particular part of the river delta once every 5 years, our environmental roulette wheel may have five times as many black slots (no flood) than red slots (flood). Each year, the wheel is spun, but each year, the result is independent of the previous spin or last year's result. What we would like to do at the beginning of each monsoon season is to use information that would change the proportion of red and black slots for a particular forthcoming period of time, month or season. The goal is to develop information that will bias the number of coloured slots by using quantitative probabilistic weather and climate-prediction models, so that user communities can place ‘informed bets’ on how to manage their resources. By attempting to determine the future state of the environment, weather and climate models provide information that reduce or increase the odds of an extreme event occurring. A model simulation commences at some prescribed time with initial data that describes to the best of our knowledge the state of the atmosphere, ocean, land surface, etc. But there are two problems associated with numerical weather and climate models.

— *Model error.* All models possess bias. Using the roulette analogy, one model may have the propensity to tilt towards more red slots than black, while another model tilts towards more black slots. Model errors arise from formulations that attempt to replicate continuous laws of physics and thermodynamics in point-wise space, different methods of numerical solutions, and the manner in which processes occurring on scales smaller than the finite elements of the model are taken into account.
— *Uncertainty in initial conditions.* A model must be provided with a set of initial conditions that define the state of the global weather or climate system at a given time. The state of the climate system is not known exactly, and small differences between the real state (which is never known) and the approximate state may lead to large nonlinear error growth in time [18,19].

Clearly, the prediction tools that we possess are imperfect because of both model bias and sensitivity to uncertainties in initial conditions. The most important question becomes: with the uncertainty associated with imperfect models and poorly defined initial conditions, can a credible forecast be provided and used to reduce vulnerability and risk?

There are a number of techniques that can be used to characterize model forecast uncertainty and minimize error in a prediction. First, one can use the predictions of many models with the same sets of initial conditions to produce a multi-model mean. The assumption is that each model has different biases that are randomly distributed among the models that ‘average out’ in producing the multi-model mean. This is a relatively weak assumption as some errors, especially those associated with the parameterization of sub-grid processes, are common to many models.

Sensitivity to initial condition error and the resulting nonlinear error growth may be assessed through making incremental changes to the model initial conditions (e.g. [20]) or to the parameters that define the representation of sub-grid processes, and running the model many times with their perturbed initial conditions. For example, the European Centre for Medium Range Weather Forecasts (ECMWF) runs 51 ensemble members during each forecast cycle [21]. These forecasts, run twice per day out to 15 days produce a swath of forecasts that tend to diverge with time. While the mean of all of the ensemble members provides some statistical information about the evolution of weather or climate, it is the divergence of the ensemble members that provides the most important information about the predictability of the future state of the environment.

**Figure 2.**
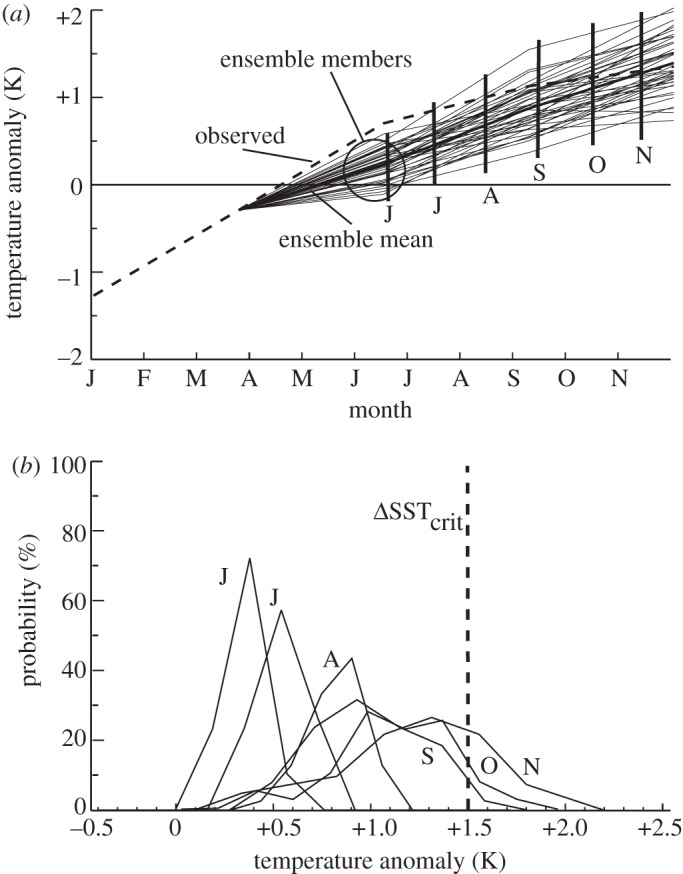
Example of an ensemble forecast from the ECMWF system 3 coupled ocean–atmosphere climate model [22] predicting the evolution of the mid-eastern equatorial Pacific Ocean sea-surface temperature (SST) anomaly (deviation from climatology). Positive (negative) anomalies indicate the advent of an El Niño (La Niña). (*a*) Evolution of the 41 ensemble members initialized in April 2009. The spread of the ensembles allows the determination of the probability of an anomaly of a particular magnitude. Vertical lines indicate the spread of the ensembles for June (J), July (J), … November (N). (*b*) Probability density functions (pdfs) of the SST anomaly at the end of each forecast month. With increasing lag, the pdf moves progressively to warmer temperatures. The probability of the SST exceeding some critical value (ΔSST_crit_) is discussed in the text.

Model bias and ensemble spread tell us what we do and do not know about the state and future of the system we are trying to predict. But given that uncertainty, the ensemble spread of the weather or climate forecasts can also be used to provide an assessment of the probability of an event of a particular magnitude occurring at some time in the future. Figure 2 presents an example of an ensemble forecast from the ECMWF system 3 coupled ocean–atmosphere climate model [22] initialized in April 2009. It shows predictions of the mid-eastern equatorial Pacific Ocean sea-surface temperature (SST) anomaly or the deviation of the SST from climatology. The SST anomaly in this area of the equatorial Pacific Ocean (160^°^ E–150^°^ W, 5^°^ N–5^°^ S, known as the Niño-4 region) is a measure of whether or not there will be an El Niño or La Niña or neutral conditions. The ensemble members provide an estimate, at some time in the future, of the probability of the occurrence of an event of a particular magnitude (figure 2*a*). The individual ensemble simulations can be used to form a probability density function (pdf) of the regional ocean temperature (figure 2*b*). Each ensemble member represents an equally likely solution, but the clustering of the ensemble members provide an estimate of the probability that anomalous SSTs of a certain magnitude will be exceeded.

Once the probability of an event has been estimated, one can define risk as

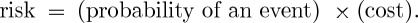

Altering the probability of a flood, for example, would require major infrastructure investments. However, risk can also be reduced by minimizing the cost associated with the event. For example, the probability of an extreme event may be very small, but the cost of the occurrence of such an extreme event may be very large, thus constituting a high risk. For example, experience may suggest that if the Niño-4 SST anomaly exceeds more than 1.5 K (a critical value; ΔSST_crit_), there is enhanced probability of higher than average precipitation in California. Using the 2009 example, it is possible to compute from figure 2*b* that there is a 1 per cent probability of the SST anomaly exceeding 1.5 K in September, 5 per cent in October and rising to 20 per cent in December. Depending on the historical cost of such events, it is possible to determine whether actions aimed at mitigating damage are worthwhile. Based upon the cost involved in earlier periods of enhanced precipitation, does the risk of occurrence justify the expenditure of alerting emergency services, clearing streambeds and planning for the evacuation of landslide-prone areas? If so, the cost can be reduced and, thus, the risk minimized.

The notion of ‘cost’ is relative. The probability of occurrence of an event in one part of the world may be the same, but the consequences (relative cost) may be very different. Along the coast of the United States, the probability of the landfall of a hurricane of a particular magnitude at some time in the future may be the same as the one occurring in the Bay of Bengal. But in the United States, transportation and communication infrastructure will lower the cost in human lives, so that the major loss will be in personal belongings and property. However, in the developing world, a hurricane of the same magnitude will invoke a much higher cost in human life because evacuation is more difficult. Absolute economic loss in Bangladesh may be smaller because intrinsic values of property and infrastructure may be lower than those for the developed world. But in terms of per capita wealth, the losses may be huge and impoverishing.

In summary, the quantitative determination of risk allows a rational determination for developing adaptation and mitigation actions to reduce costs of the user group concerned.

## 3. Adaptation to current risk of extreme events

We use as an example of short-term adaptation to risk by considering flooding in Bangladesh. The catastrophic Bangladesh flooding of 1998 prompted the United States Agency for International Development's Office of Foreign Disaster Assistance (USAID-OFDA) to fund an exploratory project (Climate Forecast Applications to Bangladesh; CFAB^1^) with a primary goal of providing advanced warning of flooding in Bangladesh on 1–10 day time scales. The presumption was that extended range stream-flow and precipitation forecasts would be of great value in the densely populated and heavily farmed river basins of Bangladesh. CFAB decided early to issue probabilistic forecasts to support risk management strategies to be developed. Before the CFAB project, the time horizon of stream-flow forecasts over much of the developing world, including Bangladesh was 2 days. This limited horizon is far too short a period for villagers and farmers to take effective mitigatory actions.

**Figure 3.**
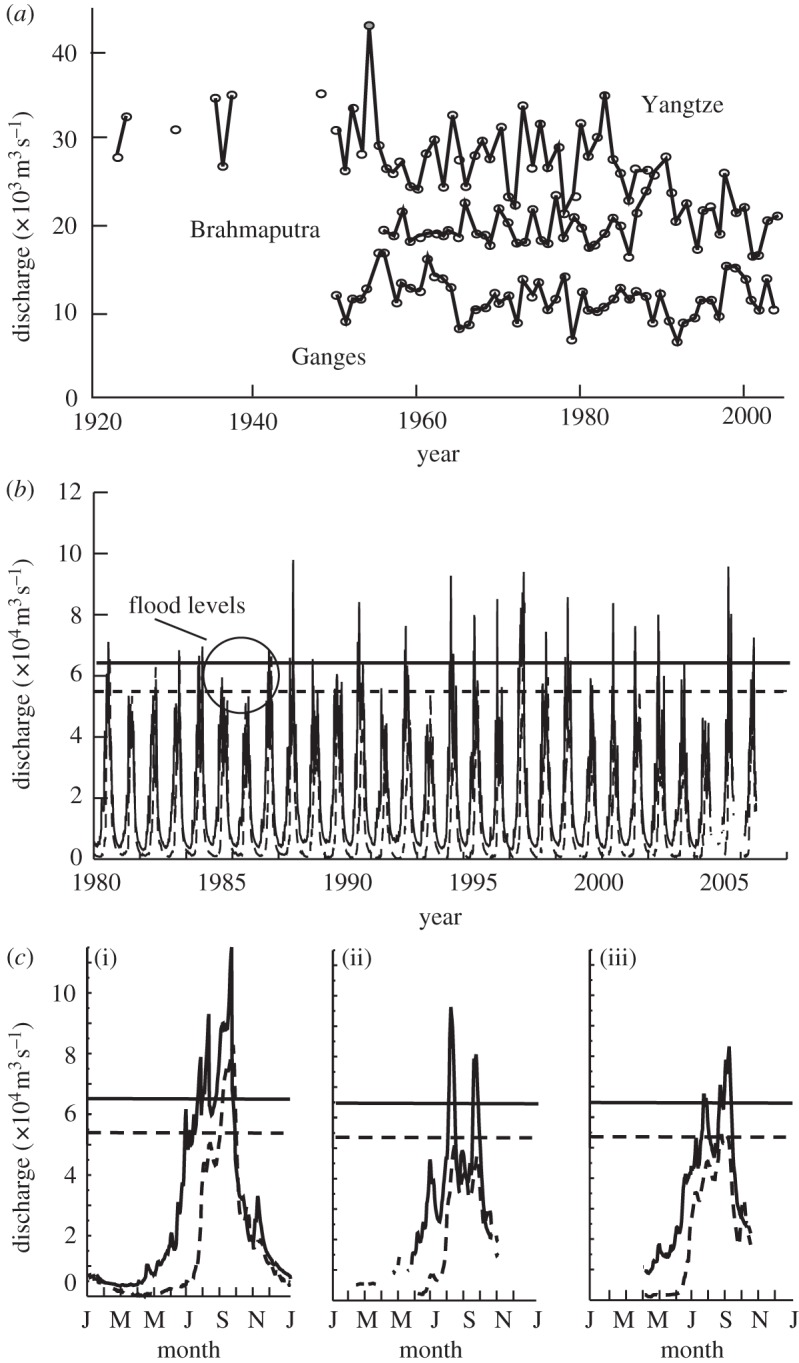
Long-period data for rivers in the developing world are relatively rare. Staging stations at the Hardinge Bridge on the Ganges, Bahadurabad on the Brahmaputra and Datong on the Yangtze (see figure 1) are exceptions. (*a*) Annual river discharge for the three rivers. The Brahmaputra and the Ganges data are obtained from the Bangladesh Flood Forecasting and Warning Centre (FFWC) and the Yangtze data from the National Center for Atmospheric Research (NCAR). (*b*) Time sections of the daily Ganges (dashed) and Brahmaputra (solid) inflow into Bangladesh for the period 1980–2009. Flood levels are shown as horizontal lines. (*c*) Details of the Ganges and Brahmaputra inflow into Bangladesh for the flood years (i) 1998, (ii) 2007 and (iii) 2008.

*Data.* Ganges and Brahmaputra River discharge data (figure 3) dates back to the 1950s. The monthly mean Yangtze River stream-flow data at the Datong station^2^ is added here for later reference. Each of these three staging stations were chosen close to the river mouth to represent the impact of basin-wide precipitation (figure 1). Mean annual values of river discharge at these points are plotted in figure 3*a*. Daily data, necessary for submonthly forecasts, are not available for the Yangtze. Figure 3*b* provides a description of the daily record of the Ganges (Hardinge Bridge) and the Brahmaputra (Bahadurabad) stream flow collected between 1980 and 2009. Periods when the flood levels are exceeded are apparent in both river basins. The horizontal dashed lines and solid horizontal lines represent the flood levels of the Ganges and Brahmaputra. River discharge for 1998, the year of the ‘century’ flood, 2007 and 2008, the first 2 years of fully operational CFAB forecasts, is shown in figure 3*c*.

An estimate of the probability of flooding exceeding particular durations for each month can be calculated from the historical Ganges and Brahmaputra discharge data (table 1). Overall, there is a 35 per cent chance of a 1 day flood event along the Brahmaputra in any particular year. This probability is reduced to 25, 20 and 8 per cent for 3, 5, and 10 day flooding, respectively. A flood with a 10 day duration has a 13 year return period. Later, we will see how these probabilities may change relative to a wide range of future climate-change scenarios.

**Table 1.** The climatological expectation of flooding of a prescribed duration occurring in a given month in (*a*) the Brahmaputra and (*b*) the Ganges in a given month. For example, there is a 15% chance of a more than 3 day flood occurring in July in the Brahmaputra. Return periods in years are shown in parentheses.

	Jun		Jul		Aug		Sep		annual	
	%	yr	%	yr	%	yr	%	yr	%	yr
(*a*) Brahmaputra										
>1 day	2	52	23	4	12	9	8	13	35	3
>3 day	2	52	15	7	8	13	8	13	25	4
>5 day	0	—	12	9	6	17	6	17	20	5
>10 day	0	—	4	26	2	52	2	52	8	13
(*b*) Ganges										
>1 day	0	—	0	—	14	7	21	5	24	4
>3 day	0	—	0	—	12	8	17	6	21	5
>5 day	0	—	0	—	10	10	12	8	16	6
>10 day	0	—	0	—	9	12	7	15	10	10

**Figure 4.**
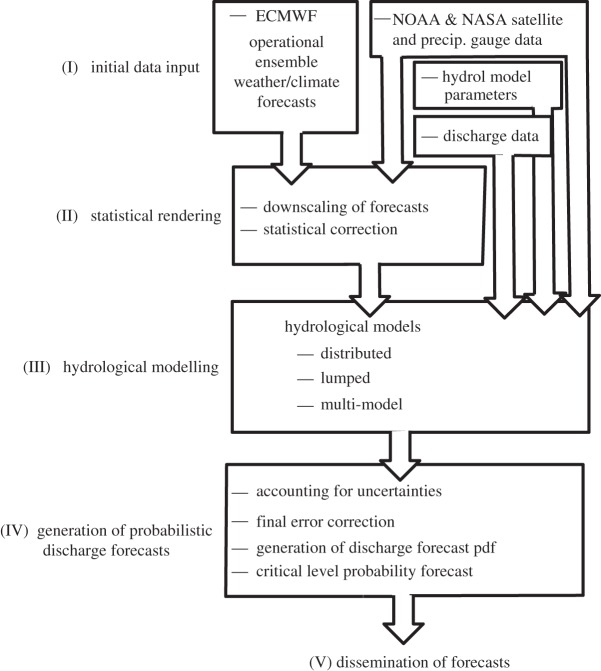
Five major steps of the CFAB probabilistic forecast scheme. The basis of the model is the Environmental Prediction Scheme (EPS; [21]) quantitative precipitation (precip.) forecasts (step I) that are statistically corrected using satellite precipitation data (step II). The adjusted precipitation forecasts, together with meteorological and initial discharge data initialize a suite of hydrological models (step III). Step IV provides a final statistical rendering. The models are then disseminated (step V) to user communities. Adapted from Hopson & Webster [23] and Webster *et al*. [24]. NOAA, US National Oceanic and Atmospheric Administration; NASA, US National Aeronautics and Space Administration.

*Model bias correction.* Figure 4 provides a flow chart of the basic composite model developed to provide probabilistic forecasts of flooding [23,24]. Overall, the forecast system incorporates traditional hydrological models within a probabilistic meteorological framework. The quantified precipitation forecasts (qpfs) come from an ensemble of ECMWF qpfs arising from perturbed initial conditions.

One aspect that needs to be mentioned is the bias correction of the ECMWF qpfs (step II, figure 4). This Bayesian correction uses a merged precipitation product from satellite observations and a quantile-to-quantile (q–q) correction at each grid point in each basin (see [23,24] for details). The virtue of the technique is that it forces each ECMWF ensemble member at each 0.5×0.5 grid point to be adjusted statistically from the cumulative distribution function of the associated satellite/gauge data at the same point. Finally, it is important to note that the q–q technique ensures that the forecasts produce the same climatological rainfall distribution as the observations, including the number of ‘no rain’ events as well as heavy rainfall events.

**Figure 5.**
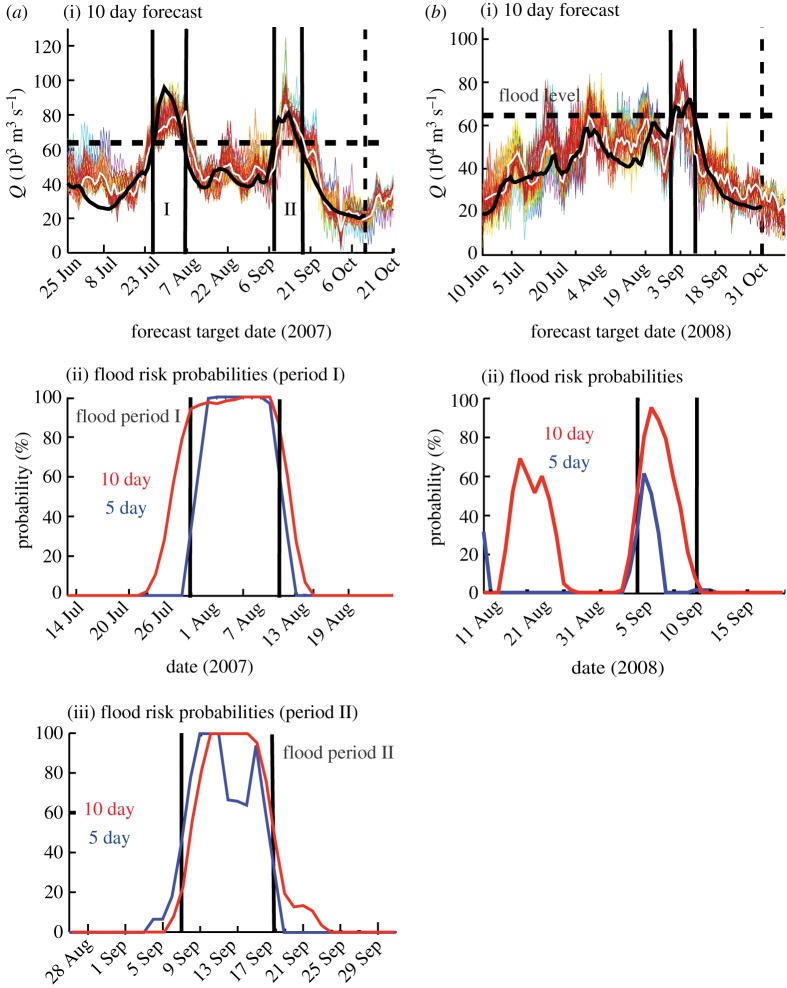
The three left-hand panels in (*a*) refer to forecasts of Brahmaputra river discharge in 2007, while the two right-hand panels in (*b*) refer to Brahmaputra flow in 2008. *a*(i) Summary of the 10 day Brahmaputra forecasts for the entire summer plotted against the forecast target data. *a*(ii) Details of the period 13 July–19 August around the flood event I 27 July–6 August 2007 and 28 August–30 September for flood II 7–17 September. *a*(ii)(iii), Flood exceedance probabilities for floods I and II (27 July–6 August 2007 and 7–17 September, respectively). *b*(i) Same as (*a*), except for 2008. *b*(ii) Shows flood exceedance probabilities for the 5–10 September period, 2008. Adapted from Hopson & Webster [23] and Webster *et al.* [24].

*1–10 day flood forecasts.* An example of the application of probabilistic environmental prediction is shown in figure 5. 10 day ensemble forecasts of the Brahmaputra River discharge at Bahadurabad are plotted against the forecast target date for the entire 2007 summer period. Two flooding events occurred (labelled I and II) during July and September, but no Ganges flooding (see [24] for details). Both flood events were forecast 10 days ahead quite accurately, both in the timing of the onset of the floods and their duration. The observed discharge (the verification of the forecast) and matching the time of the forecasts is shown as a solid black line. The ensemble mean of the 10 day forecast appears as a white line. Estimates of flood risk at 5 and 10 day horizons, calculated from the spread of the plumes, are shown in figure 5*b*(i,ii). Both the 5 and 10 day forecasts provided a very high probability of flooding at the correct time and an accurate estimate of the duration of the flood. Forecasts for the other years of the Ganges and Brahmaputra 2004–2009 period are described in Webster *et al.* [24], along with a detailed assessment of skill.

*Development of adaptation strategies.* In 2007 and 2008,^3^ a number of flood-prone unions (equivalent to counties) along the Brahmaputra and the Brahmaputra–Ganges were chosen as test bed locations for applications of the flood forecasts (see fig. 1*c*, [24]). Advanced notice of each of the impending floods was communicated by the FFWC to the unions and villages within the union by a planned cell-phone network and communicated within the villages by a series of flag alerts. In each union, government agriculture extension personnel and village leaders had been trained by CFAB partners (CEGIS and ADPC) to understand and interpret the forecasts in terms of local references and landmarks so that the inundation could be communicated unambiguously to the villagers [24].

Early in the process, local officials had acknowledged that 10 day forecasts were optimal, providing a reasonable lead time for people to make agricultural adjustments and property decisions. In addition, such advanced warning would also allow regional professionals time to coordinate local efforts, to suggest to agricultural dealers to hold off on the sale of seeds and pesticide, and to offer advice to farmers, fishermen and agricultural dealers [25]. Communities were vitally interested in when the flooding would occur, what height the flood level would be, and how long the flood level would be exceeded. This last metric is of considerable concern since inundation greater than about 10 days means that there would be little chance of any return on crop planted before the flood.

A National Disaster Emergency Response Group planned the overall emergency response and logistics for pre-flood preparedness and post-flood relief. Agencies with local representation (e.g. Department of Agricultural Extension) prepared rehabilitation plans in advance for regions of high vulnerability. With the forecast of an impending flood, communities were advised to ‘wait, watch, worry and work’ [24,25]. Evacuation assembly points were identified with adequate communication and sanitation facilities. In the vulnerable regions, defined by the forecasts, fisheries were protected by increasing the height of the retaining netting around the fishponds. Suggestions were made about harvesting crops early, ahead of the impending flood or to delay planting. Families were advised to store about 10 days worth of dry food and safe drinking water, as relief would not be forthcoming until at least 7 days after the cessation of the flood. In addition, cattle and poultry, crop seed and portable belongings were to be secured in safe locations such as on road embankments. Of particular concern were the people of the river islands (or chars) farmed by the poorest of the poor that are rapidly engulfed by rising water. Plans were made for the rapid deployment of manual and mechanized boats for evacuation. With the normal 2–3 day FFWC forecast, these extensive plans would have been impossible to implement; the 10 day forecasts produced by CFAB enabled adequate time to prepare properly for the floods [24].

*Assessment of economic value of probabilistic forecasts.* Following the floods, ADPC [25] assessed the utility of the 1–10 day forecasts, together with a cost–benefit analysis, by interviewing over 100 households. Although limited in scope, the survey indicated that there were substantial financial benefits in using the flood forecasts. It was estimated that the average savings for each household involved in fisheries was equivalent to US$130, and in agriculture, it was US$190. The greatest savings per household were from the protection of livestock (US$500 per cattle) and household assets (US$270 per household). Given that the average income in Bangladesh is approximately US$470 per year and that 50 per cent of the population exists on less than US$1.25 per day [26], the savings were substantial in the flooded regions in terms of man-years of labour. For example, the loss of one cattle would require the equivalent of 2 man-years of labour for replacement. The report concludes that the forecasts were accurate, timely and well used in the pilot unions.

The utility of the forecasts is best summed by a statement made during the ADPC evaluation following the 2008 floods [25]. It also illustrates how the forecasts were incorporated into daily village life. As described by the Imam from the Mosque in Koijuri Union of Sirajgong District in Bangladesh: ‘We disseminate the forecast information and how to read the flag and flood pillar to understand the risk during the prayer time. In my field, T. Aman was at seedling and transplanting stage, I used the flood forecast information for harvesting crops and making decision for seedling and transplantation of T. Aman. Also we saved household assets.’ (S. H. M. Fakhruddin 2010, personal communication.)

## 4. Adaptation to longer-term changes in extreme events

Three of the great rivers of South and East Asia, the Ganges, Brahmaputra and Yangtze, currently support 14 per cent of the human population on the planet (figure 1), and are centres of thriving agrarian societies. During the next century, this proportion of global population living in these river basins is expected to stay relatively constant [27] supporting, in total, populations rising from 0.8 billion to 1.5 billion.

Relatively strong relationships occur between total Indian rainfall and the El Niño Southern Oscillation (ENSO) (e.g. [28–30] on interannual and decadal periods, although this relationship has waned during the last few decades [31–36]). On longer time scales, Parthasarathy *et al.* [37,38] suggest that there has been no significant long-term trend in the All-India rainfall index^4^ during the last 100 years, although there seems to be a weakening of the South Asian summer monsoon since the 1970s [40,41], though changes vary regionally [42]. With respect to Ganges and Brahmaputra floods, Jian *et al.* [43] found precursor relationships for Ganges discharge related broadly to the ENSO. The seasonal Brahmaputra discharge, highly predictable at short forecast horizons [23,24], appeared to be only weakly linked to preceding SST anomalies, either remotely or regionally. However, Brahmaputra discharge was found to be strongly and positively simultaneously correlated with simultaneous Indian Ocean SST anomalies [43].

In a changing climate, it is important to assess the future distributions of the frequency and severity of flooding in delta areas such as Bangladesh. A number of studies have considered the impact of a doubling of CO_2_ on the monsoon circulation [44], on flooding [45–50] and on drought [51]. We attempt here to determine the frequency, duration and severity of flooding in the Brahmaputra–Ganges delta during the next century using a selected number of climate model simulations. Specifically, we wish to determine whether the probability of flood and drought will change from the twentieth to the twenty-first centuries. A major concern is the availability of adequate fresh water during the next 100 years, which is a function of both the rainfall as well and the population increase.

*Methodology.* We restrict our investigation to three main river basins: the Brahmaputra, Ganges and the Yangtze, based principally on the length of available data (figure 3*a*). To assess possible changes in flood and drought risk, we have hydrological data for the last 50–60 years.

In addition to the river flow data, we use the Intergovernmental Panel on Climate Change (IPCC) fourth assessment report (AR4) [52] and coupled model intercomparison project phase three (CMIP3) simulations [53] to obtain estimates of future river flow. Most of the 25 coupled ocean–atmosphere models were run in an ensemble mode for a three century period: the pre-industrial period, the twentieth century (in which historical values of greenhouse gas (GHG) concentrations were incorporated) and the next 100 year using projections of GHG concentrations. These GHG concentration projections are made relative to a set of scenarios (described in the Special Report on Emission Scenarios: SRES IPCC [54]). There are three basic families of SRES.

— GHG concentrations are assumed to hold constant at 2000 levels. We refer to this as the GHG2000 scenario.
— The A-family set of scenarios that assumes rapid economical and technological growth with an increasing population in the first half of the twenty-first century and a decreasing rate of growth in the second (A1) or a continuous growth rate of population throughout the century (A2). The A-family projections range between CO_2_ concentrations of 700 and 1000 ppm by the end of the century.
— The B-family of scenarios follow the A-family, except it is assumed that the economical structures of the planet are more environmentally sensitive, but with different population growth rates (B1 and B2). The A1B scenario assumes similar growth to A1, but that there is a greater spread of energy production among non-fossil choices. Typical CO_2_ concentrations for the B-family are in the range 500–600 ppm by the end of the twenty-first century.

Here, we consider four scenarios: GHG2000, B1, A1B, A1 and A2, thus spanning the full IPCC SRES range.

A cautionary note should be made regarding scenario uncertainty. This uncertainty implies that it is not possible to formulate the probability of occurrence of one particular outcome. A scenario is a plausible but unverifiable description of how the system and/or its driving forces may develop in the future. Thus, scenarios should be regarded as a range of discrete possibilities with no *a priori* allocation of likelihood.

The IPCC AR4 notes that there is much less overall agreement among models regarding changes in precipitation between models than with temperature changes (e.g. [55,56]). As river discharge depends critically on regional precipitation, it is clear that a different approach is needed in assessing future river flow. The approach has two components: model ‘culling’ and the use of a Bayesian bias reduction technique.

*Model culling.* A critical problem is how to choose which model or combinations of models from the CMIP-3 suite. Is the appropriate choice an average of all models and their families of ensembles to produce a multi-model, multi-ensemble mean as adopted by the IPCC? There are some benefits to this choice, as random error will tend to be eliminated by the averaging process. But there is a downside too. Each model possesses regional systematic biases so that not all models will produce realistic fields of precipitation in all river basins, even for the present era.

We have developed a two-step process of minimizing systematic error in the models.

— The models are rated relative to their ability to simulate the annual cycle of precipitation in the twentieth century in terms of magnitude and phase in a particular basin. This criterion is based on the observation that the three river basins have strong annual cycles with a summer precipitation maximum accounting for the majority of river flow.
— The annual precipitation of the three river basins are related to the phase of the ENSO phenomena [57] that in turn is reflected in the river discharge. For example, the Ganges has a larger discharge during a La Niña year and less in an El Niño year. The discharge is marginally greater during a La Niña year and considerably less during El Niño.

We choose these two selection criteria on the basis that unless these most basic physical ‘fingerprints’ are found in the model simulations, each related to fundamental forcing (i.e. the annual cycle of solar radiation and the largest SST anomalies), then it would be unlikely that a reasonable estimate of river discharge can be determined for the future. Of the two discriminators, the most important is the ability of the model to possess an appropriate annual cycle in precipitation.

**Figure 6.**
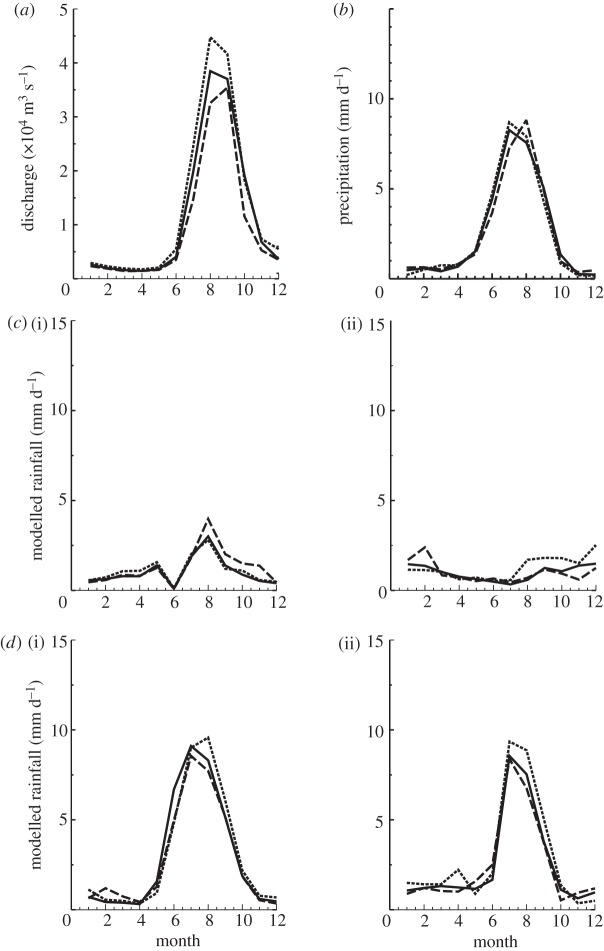
Culling of the IPCC AR4 models based on the precept that models that simulate the present era monsoon precipitation the best are most likely to perform better in estimating future monsoon precipitation. (*a*) Annual cycle of the Ganges River flow into Bangladesh (10^4^ m^3^ s^−1^) measured at Hardinge Bridge. (*b*) Same as (*a*), except for rainfall (mm d^−1^) averaged over the Ganges catchment area upstream of Hardinge Bridge. (*c*) Examples of models that failed to replicate the annual cycle of precipitation over the Ganges catchment shown in (*b*). These models were (i) the Beijing Climate Center Climate model (BCC-CM1) and the Institute Pierre Simon Laplace climate model (IPSL cm4). (*d*) Two models that did replicate the annual cycle of annual precipitation shown in (*a*). These are (i) the Max Planck Institute ECHAM5 climate model and (ii) the UK Meteorological HAD1 climate model. In all figures, El Niño years are dashed lines, La Niña years are dotted lines and normal years are solid lines.

Examples of accepted and dismissed models based on the annual cycle and the ENSO cycle are shown in figure 6. Figure 6*a*,*b* shows the observed river discharge and spatially averaged precipitation for the Ganges River basin for El Niño and La Niña periods defined by a ±1 s.d. of central-eastern Pacific Ocean SSTs. Examples of CMIP-3 models not fulfilling these basic criteria are the Beijing Climate Center Climate Model (BCC-CM1; figure 6*c*(i)) and the Institute Pierre Simone Laplace (IPSL cm4; figure 6*c*(ii)). Examples of models fulfilling the criteria are the German Max Planck Institute-ECHAM5 (figure 6*d*(i)) and the UK Meteorological Office UKMO-HAD1 (figure 6*d*(ii)). Of the total of 25 models, 13 passed the two tests for the Yangtze, 14 for the Ganges and 16 for the Brahmaputra.

*Minimization of model bias.* For those models that pass the two criteria, we need to apply a new mapping technique aimed at reducing model bias [57]. The system adopted is similar to the q–q technique of Hopson & Webster [23] mentioned earlier in the paper for the short-term flood forecasting. The Bayesian technique maps multi-model precipitation estimates obtained from the twentieth century simulations with observed river discharge for each of the three rivers. Essentially, a q–q method maps the model-space precipitation and the observed-space discharge of a particular river for each accepted model.

**Figure 7.**
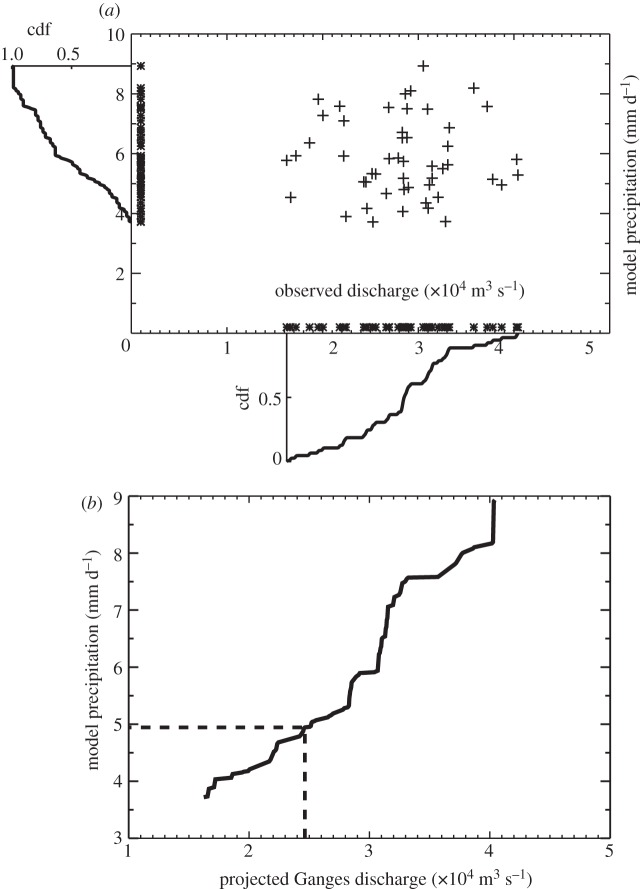
Reduction of model bias for models that passed the acceptability criteria. The biases are reduced by (*a*) performing a q–q mapping between modelled precipitation and observed river discharge and (*b*) computing a mapping index. The procedure is quite similar to the Bayesian system used in the 1–10 day prediction scheme [23,24].

Cumulative density functions (cdfs) of both discharge-space and model ensemble member precipitation-space are calculated (figure 7*a*). Common overlap periods are chosen for both the observed discharge and the simulated rainfall to ensure that the same GHG domain is sampled. The observed discharge from a particular basin is separated into *N* equally spaced sequential intervals. The model rainfall for the particular basin is also divided into the same number of intervals. An accumulated quantile value (cdf from 0 to 100) is calculated by determining the percentage of data points that are less or greater than a particular value. Then, the quantile points in model-space are mapped to the same quantile points in the observational-space (figure 7*b*). Thus, the observed 24th discharge quantile of Yangtze River discharge (e.g.) is related to the 24th modelled precipitation quantile generated for that period. This mapping technique removes, in essence, the model-to-model variability between the selected models.

The mapping index is constructed using the twentieth century simulated precipitation and observational river discharge (or gridded precipitation), both of them under the same influence of the twentieth century GHG radiative forcing. In addition, all four future SRES experiments are initialized directly from the ending state of the climate of the same GHG2000 experiment as defined separately by each model. As no other dynamic or thermal adjustments or forcing is used (other than the assumed growth of GHG concentrations under the various scenarios), it seems reasonable to apply the same ensemble-specific precipitation index to the four SRESs to construct the future river discharges. The variability of the mean river discharges is then most probably attributable to the different SRES scenarios.

Another advantage of the q–q technique comes from the monotonic non-negative slope of the mapping index profile that ensures a larger precipitation always corresponds to a larger river discharge. This index is also nonlinear, so that excessive data crowding or an isolated point on one side of the interval will not cause a twisting of the mapping. The index is independent for each ensemble member of a model, so that the variability and the mean value of the reconstructed discharge are also independent. Furthermore, the index can be extended with linear interpolation on the ending points in case the future modelled precipitation output lies outside the current observed and modelled space.

**Figure 8.**
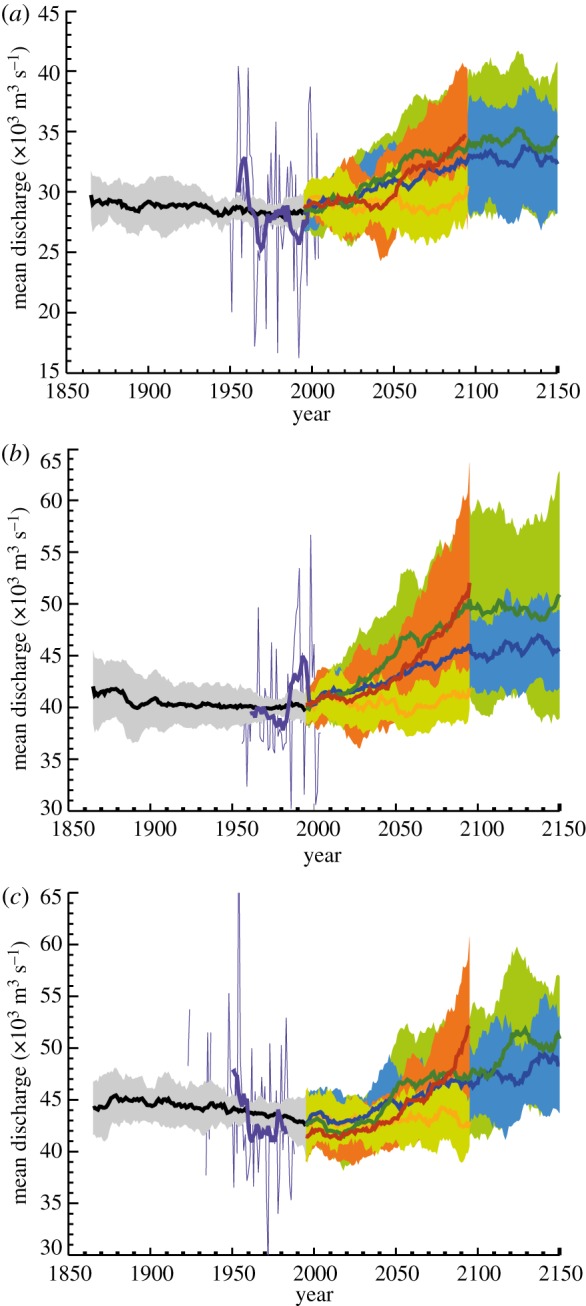
Simulated discharge (×10^3^ m^3^ s^−1^) over the next century of the (*a*) Ganges, (*b*) Brahmaputra and (*c*) Yangtze Rivers at the Hardinge Bridge and Bahadurabad in Bangladesh and Datong in China, respectively, for a range of SRESs, including the scenario when the CO_2_ concentrations are held at the 2000 level. The multi-model ensemble means are shown as the bold lines. The twentieth century simulations are in black. The grey surroundings denote ±1 s.d. The observed fields for the three rivers are in purple, and show greater interannual variability than the twentieth century simulated fields. The twenty-first century SRES fields are shown (together with ± 1 s.d.) as yellow (GHG2000), blue (B1), red (A2) and green (A1B). With the exception of GHG2000, all the scenarios show increasing mean discharges and increasing variability with time.

*Flood risk probabilities in the twenty-first century.* Figure 8*a*–*c* shows the results of the analysis of the qualified models following the q–q mapping for the Ganges, the Brahmaputra and the Yangtze for each of the four emission scenarios from the mid-nineteenth century through to the twenty-first century. The ‘plumes’ represent the evolution of each of the sets of ensembles of the accepted models, colour coded relative to the particular SRES. The bold solid lines show the evolution of the multi-model mean and the plumes represent ±1 s.d. from the mean. In general, the fixed CO_2_ concentration (GHG2000) shows a relatively constant mean value and about the same variability about the mean as during the twentieth century. This is a heartening result since it shows that this multi-model ensemble does not undergo model drift or dispersion. This result allows a more confident interpretation of the other experiments that do show change with time, such as the B1, A1B and A2, with increasing mean values and an expanding spread over the next 100 years. Given the constancy of the spread of the GHG2000 experiments, we can interpret the expanding dispersion of members as an increase in interannual variability. The largest increases occur with the A2 scenario within which CO_2_ concentrations expand to 850 ppm by the end of the twenty-first century. These results are consistent with the findings of Dairaku *et al.* [58].

Table 2 summarizes the statistics of the simulations. Discharges and standard deviations (s.d.) are listed for each river basin and SRES scenario, including GHG2000, for 25 year blocks between 2000 and 2099. Increases in discharge are projected to occur between each quarter of the twenty-first century of the multi-model simulations. In the last quarter of the twenty-first century compared with observed values for the twentieth century, one finds increases of 13 and 20 per cent for the Ganges, 15 and 30 per cent for the Brahmaputra and 10 and 19 per cent for the Yangtze, for the B1 and A32 scenarios. The s.d. for the observed twentieth century Brahmaputra discharge about the mean is 5704 m^3^ s^−1^. The s.d. for the last quarter of the twenty-first century is 9605 m^3^ s^−1^. Thus, current observed mean discharge +1 s.d. (45 731 m^3^ s^−1^) does not exceed the mean value of the last 25 years of the twenty-first century (50 413 m^3^ s^−1^).

**Table 2.** Summary of modelled, observed and projected river discharges (10^2^ m^3^ s^−1^) from figure 8*a*–*c* showing mean and standard deviation of the interannual series of river discharges (m^3^ s^−1^) from different SRES cases, the twentieth century experiment run, and the observations. Means and standard deviations for four 25 year periods (2000–2024, 2025–2049, 2050–2074, and 2050–2099) are shown.

		Ganges	Brahmaputra	Yangtze
		(10^2^ m^3^ s^−1^)	(10^2^ m^3^ s^−1^)	(10^2^ m^3^ s^−1^)
*observations*				
twentieth century 1951–2000		283 ± 57	400 ± 57	437 ± 72
*simulations*				
twentieth century 1950–2000		283 ± 59	401 ± 59	438 ± 72
twentieth century complete		282 ± 58	401 ± 60	439 ± 73
scenario	period			
GHG2000	2000–2024	291 ± 65	405 ± 63	429 ± 65
	2025–2049	290 ± 63	400 ± 61	425 ± 64
	2050–2074	285 ± 68	393 ± 60	432 ± 72
	2075–2099	290 ± 65	411 ± 67	432 ± 79
B1	2000–2024	293 ± 65	415 ± 65	433 ± 73
	2025–2049	307 ± 67	422 ± 67	447 ± 91
	2050–2074	313 ± 71	441 ± 76	454 ± 83
	2075–2099	315 ± 71	453 ± 78	473 ± 96
A1B	2000–2024	292 ± 62	411 ± 65	425 ± 70
	2025–2049	310 ± 62	435 ± 75	442 ± 78
	2050–2074	327 ± 65	475 ± 87	478 ± 101
	2075–2099	329 ± 68	498 ± 89	483 ± 100
A2	2000–2024	293 ± 65	407 ± 69	416 ± 67
	2025–2049	294 ± 64	411 ± 72	425 ± 68
	2050–2074	319 ± 66	450 ± 83	451 ± 83
	2075–2099	342 ± 78	504 ± 96	502 ± 117

**Figure 9.**
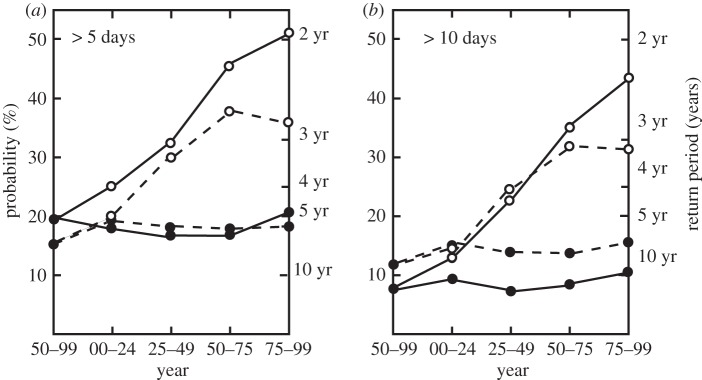
Flood probability (left ordinate) and return period (right ordinate) for floods of duration (*a*) more than 5 days and (*b*) more than 10 days for the Ganges (dashed line) and Brahmaputra (solid). Results for the GHG2000 (filled circles) and the A1B (open circles) scenarios are shown. The results for the observed period (1950–1999) are shown on the left of each panel, indicating probabilities of a more than 5 day (more than 10 day) flood of roughly 15–20% (10%) in any given year. These floods have current return periods of 5 and 10 years, respectively. Average probabilities and return periods in 25 year blocks are plotted to the right of the ordinate. For the constant GHG concentrations, the probabilities and return periods remain the same for the next century. But for the A1B scenario, probabilities increase to 40–50% for more than 5 day floods and 30–40% for more than 10 day floods. According to the simulations, more than 10 day floods may occur biennially.

If we assume that the streamflow producing a flood remains the same in the next century as in the current era, we can calculate the probability of flood duration in the future. To determine the duration of flooding in the twenty-first century, each ensemble member for a particular SRES was followed, and the duration of flood periods greater than a certain number of days was calculated. Using occurrences of flooding in the Brahmaputra and the Ganges during the second half of the twentieth century as the standard (table 1), the probability of flooding of a particular duration and return period can be calculated for each SRES considered. These results are shown in figure 9 for the Ganges and Brahmaputra. The required daily data for the Yangtze is not available, although the increase in the mean and s.d. (figure 8*c* and table 2) is similar to the Ganges and Brahmaputra so that perhaps an extrapolation of the Brahmaputra and Ganges results to the Yangtze may be an adequate approximation. Results are shown for the probabilities of 5 day and 10 day floods (left-hand ordinate) and return time of a flood of a particular duration (right-hand ordinate) plotted by year for the Ganges (dashed) and Brahmaputra (solid). The twentieth century statistics are shown on the left-hand side of the figures. In the current era, the Ganges has a 15 per cent probability of a flood with duration greater than 5 days each year. The Brahmaputra is higher at 20 per cent. Current probabilities of Ganges and Brahmaputra floods exceeding 10 days are 12 and 8 per cent, respectively. For all scenarios, except the GHG2000 (which shows steady probabilities throughout the twenty-first century), all probabilities increase substantially through the twenty-first century. For example, A1B flood probabilities increase by a factor of two for 5 day flooding and by a factor of four for the 10 day flooding. Return times of 10 day floods decreases by a factor of three by the end of the century, suggesting return periods of 2–3 years.

In summary, within the confines of confidence in the SRESs and model uncertainties, Brahmaputra and Ganges flooding can expect more frequent longer duration flooding during the coming century. According to the model results, the severity of the floods would also increase.

*Drought probability.* Determining potential river basin drought probabilities are critical for future water resource management. It may seem paradoxical to seek the probability of drought in an environment that may have an overall increase of precipitation. But, figure 8*a*–*c* suggests that there is considerable and increasing variability about the mean discharge. Thus, it is possible that drought events may become more frequent. The situation may be exacerbated by increased temperature and evaporation.

In South Asia, drought events are determined by precipitation and evaporation rate increases, and the amount needed for society, industry and agriculture [51,59–61]. We adopt the most commonly used Indian drought index introduced by Sikka [62]. It is a simple meteorological monsoon drought metric determined directly from the deficiency of seasonal rainfall. Moderate droughts and extreme droughts are defined when the quantum of the seasonal rainfall deficits are more than −1.25 and more than −2 s.d., respectively.

The probability of Ganges basin drought risk is summarized in table 3 for both moderate and extreme events in the boreal summer and winter. In the present era, based on annual mean rainfall, moderate droughts have about a 10 per cent probability and extreme events a much smaller chance of occurrence. Projections into the twenty-first century show no statistically significant change in the probability of a moderate or extreme drought, except perhaps for the A2 scenario. That is, the probability of Brahmaputra and Ganges drought remains much the same as the twentieth century probability, with perhaps a slight decrease.

**Table 3.** Meteorological drought probability (%) and return period of deficient precipitations under different IPCC SRES scenarios for the Ganges Rivers. Numbers rounded to nearest whole number. The scenarios in the twenty-first century are divided into two periods (2000–2049 and 2050–2099).

Ganges River Basin drought probability (%) and return period (in years)					
		moderate drought		extreme drought	
		(<mean −1.25 s.d.)		(<mean −2 s.d.)	
based on annual mean rainfall		%	years	%	years
observed (after 1950)		10	10	4	25
twentieth century simulation		10	10	2	65
scenario	period				
fixed GHG	2000–2049	12	9	3	34
	2050–2099	14	7	2	40
B1	2000–2049	10	10	3	49
	2050–2099	10	10	3	43
A1B	2000–2049	13	8	3	51
	2050–2099	10	10	3	47
A2	2000–2049	13	8	2	43
	2050–2099	9	12	2	41

**Figure 10.**
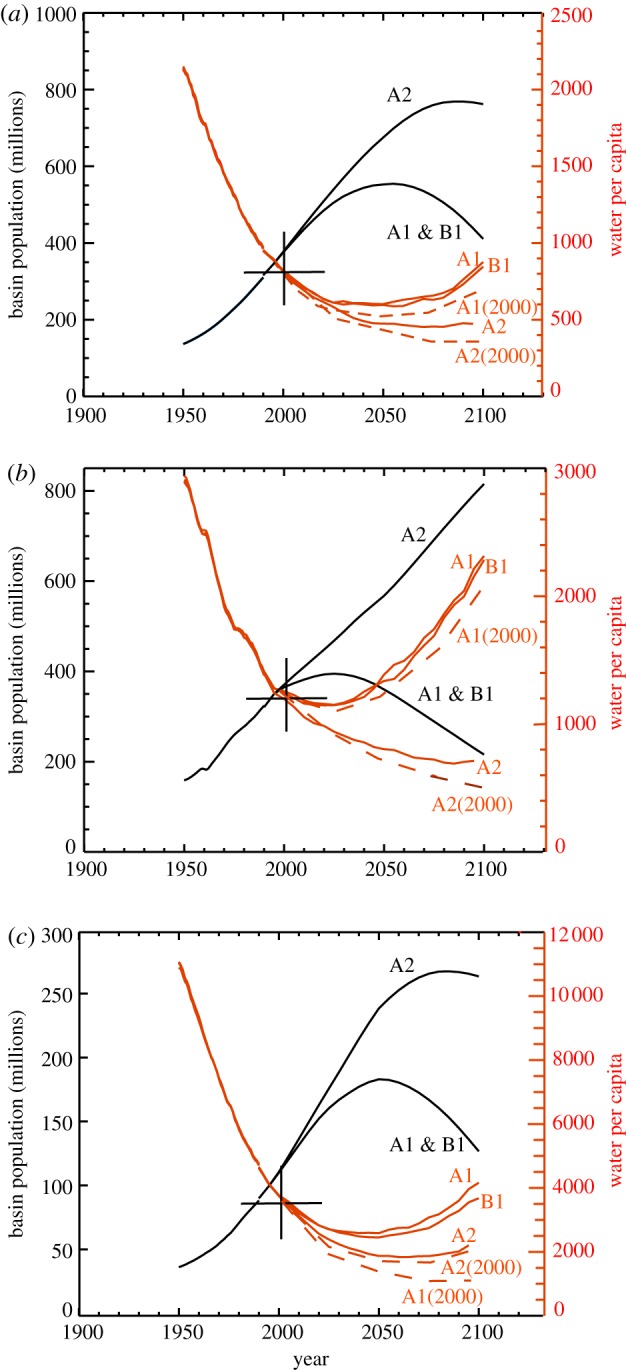
Population (black curves, left-hand ordinate) for the A2, A1, B1 scenarios and fresh water availability (discharge per population; red curves, right-hand ordinate) for the twenty-first century for the (*a*) Ganges, (*b*) Yangtze and (*c*) Brahmaputra River basins. Fresh water availability is dominated by projected population increases (solid red curves), and the increases in discharge are largely irrelevant. Dashed red curves show fresh water availability for the GHG2000 for the A1, A2, and B1 population growth rates. In the latter case, fresh water availability decreases monotonically with time.

*Fresh water availability.* Given probabilistic projections of river discharge throughout the next century and a range of the projected population growth, it is possible to make a broad-brush estimate of the availability of fresh water in each of the three basins. We calculate the total basin population based on the expected country population [27], and assume that the ratio of the basin to the entire country population would stay at the year 1990 level throughout the twenty-first century. Total basin population, river discharge and fresh water per capita are plotted in figure 10*a*–*c* for the A1, A2, B1 and GHG2000 scenarios.

Here, we equate river discharge with fresh water availability. Overall, the results of the analysis show that availability is driven almost exclusively by population growth rather than by climate change. The 20–30% increases in mean river discharge (apparent in figure 8*a*–*c*) are almost irrelevant factors in the face of projected large population increases. This is particularly obvious in the A2 case that, associated with large population increases, suggests a fresh water availability decreasing by a factor of 1.5 in all three river basins. On the other hand, basins will maintain fresh water supply per person at the current level under the A1/B1 case, based on the assumed steadying or retreating of the population in the middle and late parts of the twenty-first century. This is particularly evident in the Yangtze basin. In figure 8, it was apparent that there was a low risk of river discharge change if the GHG levels are maintained at their present levels. Assuming that the population follows the A1 or B1 trajectories, it can be seen that the fresh water depletion is greatest in each basin if there is no climate change.

This analysis on the availability of fresh water is rather simplistic because it ignores feedbacks that may occur with population if water availability per capita were to diminish. However, it provides a first approximation that may be useful for policy makers dealing with adaptation to a changing hydrological regime that includes both the impacts of climate change, no climate change and population growth.

## 5. Summary

We have considered examples of how environmental prediction allows the development of risk management and adaptation strategies to deal with extreme events that occur in the present climate era and those that may be encountered during the next century in the range of the IPCC SRESs. Strategies depend upon environmental prediction, but short-term adaptation is best handled at the community level, whereas long-term adaptation requiring major engineering innovations may have to be handled bi-laterally or multi-laterally.

We have argued that the continual exposure to extreme events (floods, droughts, tropical cyclones) affecting communities every few years places families in economic jeopardy and should be considered as an important reason leading to structural poverty. Furthermore, we have shown that some of the impacts are avoidable. Our fundamental hypothesis is that those who have experienced catastrophic loss owing to extreme events are those most adept at understanding risk and developing adaptation strategies. For this reason we took probabilistic flood forecasts directly to the village level. Indeed, ADPC's post-flood economic analysis found averaging economic savings in households and farms that were measured in units of annual income.

Webster & Hoyos [63] offered another example of how environmental prediction could have a very positive impact on the alleviation of poverty. They showed that there was strong predictability of regional rainfall on the 20–25 day time scale. From an agricultural perspective, this prediction horizon is optimal and arguably far more important than information from a seasonal forecast, which by necessity would be far less space and time specific. For example, a seasonal precipitation forecast of the All-India Rainfall Index, as produced by the Indian Meteorological Department,^5^ is not regionally specific. Even a perfect forecast of an above or below average All-India Rainfall Index provides little help to the farmer [63,64]. On the other hand, regional forecasts on the time scale of weeks, which provide relatively specific timing of precipitation events and dry periods on the spatial scale of Indian states, would allow farmers to optimize the timing of planting and harvesting. Webster and Hoyos argue that much of the crop loss in India in the very dry year of 2002 could have been avoided if 20 day forecasts had been available. Planting over much of India took place in an early wet spell that was followed by a dry spell. Hoyos and Webster's precipitation predictions forecast the length of the dry spell and suggest that if general crop planting had been delayed by 2 weeks, the impact of the disaster could have been mollified considerably.

In the developing world, there are two specific agro-hydrological regimes. These are the irrigated sectors and the rain-fed areas, the latter occupying 65–70% of all agriculture in the developing world. The advantage of irrigation is that available water in a particular sector comes from a wide collection area, so that even if a particular region has depleted rainfall, water from areas receiving greater rainfall is available for agriculture. Multi-week forecasts, such as those of Webster & Hoyos, are of great utility even in an irrigated area, allowing water resource managers to plan ahead and determine proportions of water storage used for power generation or irrigation. As irrigation water is expensive, a rainfall forecast will allow a farmer or a water manager in an irrigated area to plan out a watering strategy and optimize usage.

In a rain-fed region, there are far fewer options. The only available water is that delivered locally by rainfall or, in some circumstances, from an artesian source. In India, for example, areas of endemic rural poverty are located in rain-fed areas. Multi-week forecasts may allow an optimization of cropping in these locations, enabling the reduction of the level of poverty. The use of probabilistic environmental predications for improving food security will be the subject of a separate paper.

We also considered the role of environmental prediction for the development of adaptation strategies requiring long-term planning and large investment. These are central issues faced by governments as they plan for water resource management activities that may involve major construction (such as the development of fresh water storage and river diversion) and, at the same time, avoiding cross-border conflict. For example, China has made tentative plans to divert the Brahmaputra to irrigate the Gobi Desert region in Central Asia, a proposal that has caused great international concern in the region (see [65] for a discussion). India has been planning for the diversion of the Ganges and the Brahmaputra to allow irrigation of the currently rain-fed regions of India for a long time. These plans, too, have raised international tensions between India and Bangladesh (see [66] for a discussion). More recently, in Kashmir, hydroelectric schemes within India concern Pakistan [67]. Even if ‘spatial’ diversions of rivers do not take place and the dams are used for hydroelectricity, there may be problems associated with ‘temporal’ diversion as water is withheld for periods to optimize energy generation. During low winter flow periods, temporal diversions may have large impacts on irrigation in the lower reaches of basins. Rational decisions can only be made at national and international levels, taking into account probabilities of climate change, population growth, the sustainability of agriculture and the needs of society downstream, provides, hopefully a wide range of viable options.

Using a range of climate model simulations, population estimates and GHG growth scenarios, it was suggested that there is a high probability of increased river discharge, especially in the last quarter of the twenty-first century. Also, flood frequency at all time scales increases with reduced return period. Droughts, though, do not appear to become more frequent, except in the winter season. Based on these imperfect models and the SRES scenarios, the future ranges from no change (with GHG concentrations held at 2000 levels) to more frequent floods and a higher discharge in all three river basins (with A and B family scenarios). If the higher probability of extreme events were to eventuate, poverty will be harder to alleviate without major infrastructure investment in water management. Yet, in terms of the availability of fresh water, possibly the most critical factor facing the developing world is dominated by population growth and not climate change. Paradoxically, the greatest challenge to the three deltas in terms of fresh water availability considered occurs if there is no climate change during the next century. In summary, we have provided examples of how environmental prediction may help in the development of adaptation strategies in the developing world. The incorporation of quantitative risk assessment into the daily lives of diverse user groups can have the effect of minimizing property and income loss. It also provides a good adaptation strategy for future challenges associated with climate change and increasing population. These challenges are serious. Obersteiner *et al.* [68] notes that societies may have to contend with non-linear interactions between eco-systems and climate change. To this, we add the complexity of population growth. Unattended, these interactions could lead to ‘…. sudden upward shift in the level of climate related damages and disasters that finally result in civil unrest in some regions of the world as those societies lost their capacity to deal with the additional climate risk(s)…..’. Obersteiner *et al.* [68, p. 12].

## Footnotes

1 CFAB (http://cfab2.eas.gatech.edu) is an international consortium led by the Georgia Institute of Technology in collaboration with ECMWF (http://www.ecmwf.int/), the Bangladesh Meteorological Department (BMD, http://www.bmd.gov.bd) and the Bangladesh Flood Forecast and Warning Centre (FFWC, http://www.ffwc.gov.bd) and the Asian Disaster Preparedness Centre (ADPC, http://www.adpc.net) in Thailand.

2 Retrieved from archives at the National Center for Atmospheric Research (NCAR) ds552.1, available online at http://dss.ucar.edu/catalogs/ranges/range550.html.

3 ADPC (http://www.adpc.net/v2007/) and the Centre for Environmental and Geographic Information Services (CEGIS, http://www.cegisbd.com) are non-governmental organizations located in South Asia.

4 The All-India rainfall index (AIRI) is a measure of the average rainfall over the entire Indian sub-continent [39].

5 Forecasts at http://www.imd.gov.in and also ‘Monsoon on Line’, http://www.tropmet.res.in/~kolli/mol/Monsoon/frameindex.html.
